# Useful field of view test performance throughout adulthood in subjects without ocular disorders

**DOI:** 10.1371/journal.pone.0196534

**Published:** 2018-05-01

**Authors:** Karlijn Woutersen, Albert V. van den Berg, F. Nienke Boonstra, Thomas Theelen, Jeroen Goossens

**Affiliations:** 1 Department of Cognitive Neuroscience, Section Biophysics, Donders Institute for Brain, Cognition and Behaviour, Radboud University Medical Center, Nijmegen, the Netherlands; 2 Royal Dutch Visio, National Foundation for the Visually Impaired and Blind, Huizen, the Netherlands; 3 Department of Ophthalmology, Donders Institute for Brain, Cognition and Behaviour, Radboud University Medical Center, Nijmegen, the Netherlands; National Yang-Ming University Hospital, TAIWAN

## Abstract

Previous research has shown an age-related decline in Useful Field of View (UFOV) test performance, which measures the duration required to extract relevant information from a scene in three subtasks. However, these results are mostly based on data that may have been confounded by (age-related) ocular diseases. We examined UFOV performance in subjects aged 19.5 to 70.3 years to investigate how UFOV performance changes throughout adulthood. All subjects underwent a thorough ophthalmological examination to exclude ocular disorders. We also examined some elementary visual functions, i.e., near and far visual acuity, crowding and contrast sensitivity. We investigated whether these functions were related to age and whether they could explain a possible age-related decline in UFOV performance. The subjects (n = 41) performed very well on almost every measure and reached far better UFOV and visual acuity scores than those reported by other studies that relied on self-reported absence of ocular pathology. We did not find significant relationships between age and any of the elementary visual functions or the first two UFOV subtasks (R^2^_UFOV1_ = 0.03, *p* = 0.25; R^2^_UFOV2_ = 0.07, *p* = 0.10). However, we found an age-related decline in performance on the third UFOV subtask (R^2^_UFOV3_ = 0.36, *p* < 0.001), which was unrelated to performance on the elementary visual function tasks. Our results show that performance on the first two UFOV subtasks as well as central elementary visual functions may remain high in the absence of obvious ophthalmological pathology.

## Introduction

Many elderly people experience difficulties in their daily life activities that depend on visual functioning, such as driving, visual search and mobility. Traditional ophthalmological measures, such as visual acuity and visual field are sometimes incapable to capture these difficulties [1]. Effective use of visual information in daily life however, does not only require good quality under optimal conditions, as is the case for these measures. It also requires fast selection of relevant information from the visual field. The useful field of view (UFOV) is the area from which information can be extracted with one glance, without head or eye movements [2]. The UFOV can be quantified in several ways. For example, some tests measure the spatial extent under various circumstances, some use a fixed eccentricity and measure the amount of time necessary to perform the task and others measure the correct response rate while using fixed eccentricity and presentation time. In this report we focus on the current version of the commercially available UFOV test, which measures minimal presentation duration required for various tasks. This computerized test currently consists of three subtests. In the first subtest, only one high contrast central stimulus is shown which should be identified. In the second subtest, the subject should again identify the centrally presented stimulus, but should also locate a peripherally presented stimulus. The third subtest is essentially the same, but here the stimuli are surrounded by distractors. Every subtest measures the presentation duration of the stimuli that the subject needs to respond correctly on 75% of the trials. Note that higher scores therefore mean lower performance [3]. Many daily life activities, especially driving ability, have been associated with UFOV performance [4–6].

Edwards et al. provided normative UFOV values for subjects over 65 years and reported that performance declines with age in this group [7]. Using a slightly different task, Sekuler, Bennett and Mamelak found that performance on a peripheral localization task steadily declines from the age of 20 years onwards while performance on a central identification task remains stable until the age of 40 years after which it rapidly deteriorates [8]. In addition, the ability to perform the two tasks simultaneously gradually becomes less after 20 years of age. Declining UFOV performance may occur for various reasons. Sekuler, Bennett and Mamelak noted that the drop in performance on their central identification task corresponds to the typical onset age of presbyopia [8]. Other non-pathological changes of the eye that occur over time also reduce visual functioning and may influence UFOV performance as well. For example, increased optical density of the lens and reduced pupil size decrease contrast sensitivity [9], which in turn has been related to UFOV performance (e.g., [10], for a summary see [11]). In addition, several ocular diseases may occur with increasing frequency in elderly, such as glaucoma, age-related macular degeneration and cataract. Indeed, lower UFOV performance has been observed in patients with glaucoma and central visual field loss mostly due to age-related macular degeneration [12,13]. In addition, patients obtained significantly better UFOV scores after cataract surgery than before [14]. Thus, although stimuli used in the UFOV task are large and of high contrast, performance does depend on accurate vision to some extent.

Nonetheless, most studies investigating UFOV performance have relied on a self-reported absence of visual deficits or a low cut-off visual acuity score but did not thoroughly examine their subjects’ eyes (e.g., [4,7,15–18]). Some performed an ophthalmological examination, but included patients with ocular disorders regardless [19,20]. In addition, participants’ refraction errors were not always corrected properly with regard to testing distance. For example, Sekuler, Bennett and Mamelak noted that presbyopia may account for the age-related decline in the central task, implying they did not use age-appropriate lenses that would have resolved this condition [8]. Furthermore, in a population study of two thousand older licensed drivers, Owsley et al. showed age-related declines of UFOV scores while using habitual correction appropriate for testing distance [21]. However, their sample included subjects who suffered from various ocular disorders, including glaucoma and age-related macular degeneration. Reduced UFOV performance due to ophthalmological disorders can therefore not be ruled out in previous research. Here, we examined how UFOV performance changes in healthy subjects throughout adulthood. All subjects underwent a thorough ophthalmological screening to exclude ocular pathology.

In addition, we investigate elementary visual functions previously related with UFOV performance, i.e., near and far visual acuity, contrast sensitivity and crowding, in the same group of healthy adults without ocular pathology. These elementary visual functions have also been reported to decline with age [22–27]. As for UFOV performance, not all studies on these measures extensively screened their subjects, provided proper refractive correction adapted to test distance, or used non-truncated charts to measure visual acuity. Elliott, Yang and Whitaker showed that a carefully screened sample of subjects with optimal refractive correction could reach far better visual acuity scores when using non-truncated charts (i.e., charts that could measure visual acuities up to -0.2 logMAR) than samples that were not screened, received habitual refractive correction and/or were tested with a truncated chart [22]. Thus, our second aim was to investigate these elementary visual functions and whether they can explain a possible age-related decline in UFOV performance.

## Methods

### Subjects

We recruited 46 healthy subjects (24 males, 22 females) of 18 years and older with public advertising between June 2015 and May 2017. We screened candidates with a structured telephone interview and excluded anyone who reported a visual (5 subjects) or neurological (9 subjects) disorder, low birthweight (less than 2500g) or premature birth (pregnancy duration < 37 weeks) to rule out effects due to an underdeveloped visual system, current pregnancy or breast feeding (1 subject), contraindications for both tropicamide and phenylephrine, or for oxybuprocain. The data collection of three subjects was terminated, as previously unknown or unreported ophthalmological and neurological disorders became apparent (see Results). The study was approved by the medical ethical committee Arnhem-Nijmegen and was conducted in accordance with the declaration of Helsinki. All subjects provided written informed consent before data collection.

### Procedure

After the intake interview, participants received an extensive ophthalmological examination to investigate the presence of any visual disorder. Subjects who showed anatomical anomalies and reduced performance on a functional measurement corresponding with the anomaly, which would influence our measurements of interest, were excluded. Measures included frequency doubling technique to assess the visual field, stereopsis, funduscopy, fundus photography (Topcon TRC-50IX; Topcon Corporation, Tokyo, Japan) and optical coherence tomography imaging (Spectralis, Heidelberg Engineering, Heidelberg, Germany). In addition, we measured participants’ elementary visual functions, specifically near and far visual acuity, contrast sensitivity and crowding intensity (for details see below), to investigate age-related changes in these measures and their relations with UFOV performance. Crowding refers to a reduced performance on a visual task when other contours are in close proximity to the target stimuli [28]. Matas et al. found a relationship between UFOV3 and crowding [10], possibly due to the additional distractors surrounding the targets in this subtest. All measures were done with the participants’ current refractive correction, unless this did not correspond to an autorefractor measurement or if it was assessed more than a year before data collection. In these cases, we used the best refractive correction determined with subjective refraction just before data collection. In addition, we used age-appropriate correction for near vision tests to exclude effects of presbyopia. We measured the subjects’ performance on several visual computer tasks, including the UFOV task and a contrast sensitivity task as described below as well as a visual motion task, an auditory processing speed task and a visual speed-acuity task which will not be discussed here. To obtain objective measures of visual functioning, we measured electrophysiological responses, including pattern visually evoked potentials (pVEP) and multifocal electroretinograms (mERG) according to guidelines established by the International Society for Clinical Electrophysiology of Vision (ISCEV) [29,30]. We used the RETI-port system (Roland Consults, Stasche & Finger GmbH, Brandenburg an der Havel, Germany) together with gold electrodes with 1.5 mm DIN sockets for pVEP measurements. We averaged responses to stimuli in each of the two eyes to increase the signal-to-noise ratio. We determined latencies for the N75, P100 and N135 components with automatic peak detection software, after which we visually inspected the peaks and corrected them if necessary. In addition, we calculated the difference in amplitude between the N75 and P100 responses and between N135 and P100 components. MERG measurements were performed with Dawson-Trick-Litzkow (DTL) electrodes also with the RETI-port system. We averaged signals from all stimulated fields and both eyes to obtain one response curve per subject to increase signal-to-noise ratio. We determined latencies for the first positive and first negative peak in the signal and calculated their difference in amplitude.

### Equipment

For the computerized psychophysical tests, we used a Dell^®^ Precision T1700 PC running Windows^®^ 7 Professional with an Intel^®^ Xeon^®^ E3-1220 v3 processor (3.10 GHz), 8 GB of RAM and an AMD FirePro™ W2100 (FireGL V) graphics adapter. We used two different monitors for the tasks. The UFOV task was done at 50 cm distance with a 19” Philips 190B LCD monitor, using a resolution of 1280 x 1024 pixels and a refresh rate of 60 Hz.

The contrast task was done at 132 cm, using a 22” COMPAQ P1220 CRT monitor running at a refresh rate of 120 Hz with its resolution set to 1024 x 768 pixels. During both tasks, the subjects’ head was stabilized using a chin and forehead rest.

### Useful Field of View task (UFOV)

We used the commercially available UFOV task^®^ version 7.0.2 (Visual Awareness Research Group, Inc., Punta Gorda, FL, USA) to assess subjects’ performance on its three subtasks [3,31]. All subtasks apply a staircase procedure to measure the threshold of stimulus presentation duration at which the subject is able to perform 75% correct. During the first subtask, a full contrast stimulus (23 cd/m^2^ against a 0.13 cd/m^2^ background), either a car or a truck of 1.7 x 1.1 degrees, is presented within a white fixation box of 2.9 x 2.9 degrees in the center of the screen and the subject has to identify it. In the second subtask, the same central stimulus is shown together with a peripheral stimulus which is always a car of equal size and contrast as the central stimulus. It can occur at one of eight equally spaced locations at an eccentricity of 13.7 degrees. The subject again has to identify the central stimulus (car or truck) and also localize the peripheral one. The third subtask is essentially the same as the second one, but now the stimuli are surrounded by distractors that the subject should ignore. Distractors were presented in 3 rings at eccentricities of 4.6, 9.1 and 13.7 degrees. Subjects were encouraged to respond as accurately as they could and to guess if they did not know the answer(s). The three subtasks were always done in the order described above. Before each subtask started, participants practiced at least four trials.

### Visual acuity

Near and far visual acuity were measured monocularly with early treatment diabetic retinopathy study (ETDRS) charts (Precision Vision, Woodstock, IL, USA) [32]. Far visual acuity was measured at 4 m distance in a dark room with a light box. Subjects were allowed to continue to the next line if they had read at least three out of five letters correctly. Visual acuity (in logMAR) was considered to be the last line where subjects read at least three letters correctly. We measured near visual acuity at 40 cm distance with ETDRS 2000 series charts (Precision Vision, Woodstock, IL, USA) lighted by a MAULatlantic desk lamp (Maul, Bad König, Germany) in an otherwise dark room to create similar lightening conditions for all measures. Again, if subjects read at least three letters on a line correctly, they proceeded to the next line. Visual acuity (in logMAR) was determined as the last line where subjects could read at least 3 letters correctly.

### Crowding intensity

We measured binocular near visual acuity at 40 cm using crowded and uncrowded LEA charts (Good-Lite Co., Elgin, IL, USA; LH version C-test ratio in [33]). Subjects had to read the first five symbols on both charts. Visual acuity was defined by the last line where subjects read at least 3 letters correctly. The difference between the visual acuity in logMAR on both charts, i.e. VA_crowded_−VA_uncrowded_, determined the crowding intensity score.

### Contrast sensitivity

To increase comparability of UFOV and contrast sensitivity performance, we measured the latter with a custom-made computer task using Matlab R2013a (MathWorks^®^, Inc., Natick, MA, USA) and psychtoolbox-3 [34–36] with stimuli similar to those in the UFOV task. A small fixation circle (0.1 degrees) was shown in the center of the screen. Behind the circle, a stimulus appeared which could be either a car or a truck, as in the UFOV task, but smaller (1.0 x 0.7 degrees). The stimuli consisted of a mixture of spatial frequencies, mostly below 1.0 cycles/degree (see S1 Fig for the spectrum), and remained visible until the subject responded. The contrast of the stimuli adapted according to the subjects’ performance using the QUEST procedure [37]. This procedure uses prior knowledge on the psychometric function and performance on previous trials during the experiment to determine the most probable Bayesian estimate of the threshold. We determined the Weber contrast threshold by calculating the mode of the posterior probability density function after 100 trials.

### Data analysis

We analyzed the data with R version 3.1.2 [38]. Before performing any analyses, we centered age on the mean of the sample (47.0 years). Then, we performed univariate linear regression analyses for each elementary visual function variable, i.e. far and near visual acuity, crowding intensity and contrast sensitivity. To investigate the relationship between age and UFOV performance, we performed similar univariate linear regression analyses for all UFOV subtests scores, thus the regression formula was as follows: *UFOV* ∼ *β*_0_ + *β*_1_ * *age_c_*. Next, we added an age_c_^2^ predictor term to the regression models to investigate a possible non-linear, quadratic relationship between age and UFOV scores. The regression formula became: $UFOV∼\beta_{0}+\beta_{1}*{age}_{c}+\beta_{2a}*{age}_{c}^{2}$. The reason for adding this term is that UFOV performance is known to first increase during childhood [39] and then decrease again at older ages [7]. In addition, Weaver, Bédard, McAuliffe and Parkkari showed that age^2^ significantly better predicted UFOV scores than age [40]. Last, to examine whether elementary visual functions could explain age-effects on UFOV performance, we added these variables, i.e., near acuity and far acuity of the best eye, contrast sensitivity and crowding intensity one by one as additional predictors. That is, these regression analyses contained two predictors: age_c_ and one of the elementary visual function variables, the regression formulas were: *UFOV* ∼ *β*_0_ + *β*_1_ * *age_c_* + *β*_2*b*_ * *var_elementary–visual–function_*. Because the residuals were not normally distributed, we bootstrapped some of the regression analyses 10,000 times to obtain more reliable standardized βs using the R boot package version 1.3–18 [41,42]. We also analyzed the total (i.e. summed) UFOV scores, but those results are reported in the supplemental materials.

Due to unavailability of the contrast task and near visual acuity ETDRS charts at the initial stage of data collection, this data is missing for 9 and 5 subjects, respectively. Therefore, we imputed those values for the regression analyses to enable valid comparisons between them (multiple imputation method with the HMisc package version 3.17–4 [43] in R). We created five datasets, each with different imputed values., but we only report results of the regression analyses for the dataset of which the correlations are closest to those based on the dataset without any imputed values (results were similar for all datasets).

## Results

### Screening

**Fig 1:**
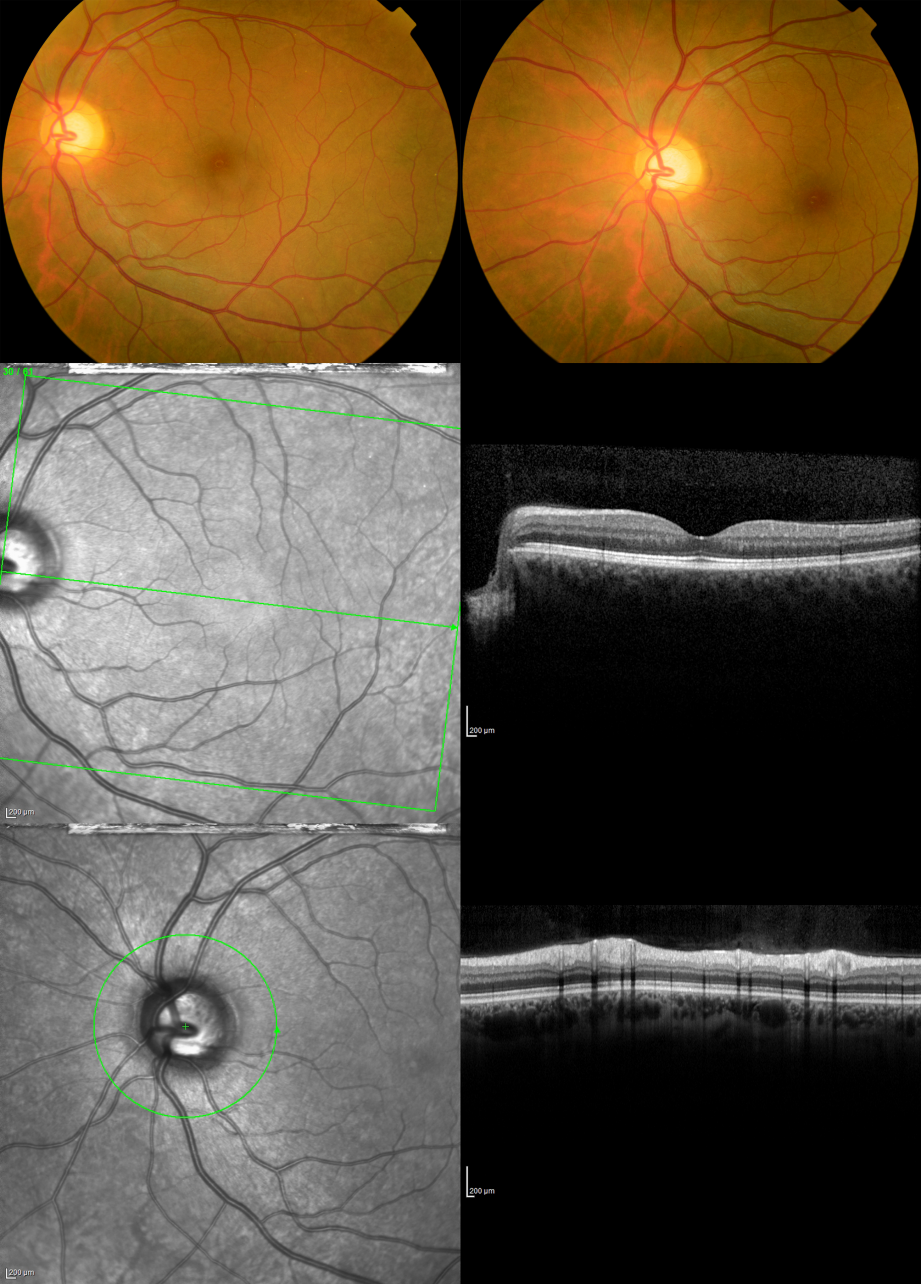
Example fundus photos and OCT scans of the left eye of a typical participant. Images were made of the posterior pole and optic disc and nerve for each eye.

All participants underwent a thorough ophthalmic screening including slit lamp examination, funduscopy and OCT imaging (Fig 1). In twenty participants’ eyes, no abnormalities were found (Table 1). The remaining 26 participants showed a variety of anomalies. These were mainly benign findings that show variability in anatomical characteristics in healthy eyes and are not pathological, for example tilted optic disc or non-sight threatening mild to moderate cataracts appropriate to subjects’ ages. However, in two participants we found severe optic atrophy with corresponding visual field defects and one participant appeared to have Lyme disease. An additional participant was unable to maintain stable fixation and one participant may not have received the right refractive correction due to a lack of cooperation during subjective refraction. Consequently, we excluded those five participants from the study.

**Table 1:** Ophthalmic screening: anatomical findings of the study participants.

Anatomical structure	Ophthalmic finding	Number of participants included in analysis (n = 41)	Number of excluded participants (n = 5)
**All**	**No abnormalities**	19	1 (excluded because of instable fixation)
**Disc**	**Enlarged excavation optic disc**	4 (both eyes)	0
	**Tilted optic disc (physiological variant)**	3 (1 both eyes, 2 one eye)	0
	**Optic atrophy**	0	2 (excluded because of optic atrophy + visual field defect, both eyes)
	**Minimal persistent arteria hyaloidea**	1 (one eye)	0
	**Peripapillary atrophy**	2 (1 both eyes, 1 one eye)	0
**Lens**	**Mild cataract**	11 (both eyes)	1 (both eyes, excluded because of Lyme disease)
**Periphery**	**Pigment shift**	1 (both eyes)	0
	**Drusen (single, small and hard without evidence of macular degeneration)**	2 (1 both eyes, 1 one eye)	0
	**Peripheral retinal scar (no absolute visual field defect)**	2 (one eye)	0
**Adnexe**	**Mild Ptosis (optic axis free)**	0	1 (excluded because of incorrect refraction at the start of measurements)
	**Dermatologic anomaly**	1	0
**Cornea**	**Age appropriate guttata**	1 (both eyes)	0

The remaining group of 41 participants had a mean age of 47.0 ± 16.0 years (range 19.5–70.3). Importantly, this group only included participants who showed subclinical findings or none at all. Their optic disc sizes were on average 1.8 ± 0.4 mm^2^ (range 0.8 to 3). MERG and pVEP curves were visually inspected and considered normal for these participants (Table 2). Near visual acuity of the best eye ranged from -0.3 to 0.1 logMAR (mean = -0.1, SD = 0.1) and far visual acuity of the best eye from -0.3 to 0.0 logMAR (mean = -0.1, SD = 0.1; Table 3). Crowding intensity varied around 0 (mean = -0.02, SD = 0.1) and Weber contrast thresholds ranged from 2 to 12 (mean = 5, SD = 2).

**Table 2:** Ophthalmic screening: Electrophysiology of subjects included in the analyses.

	min	max	mean	SD	n
**pVEP 15’ P100**_**latency**_ **(ms)**	106	151	123	10	41
**pVEP 15’ P100-N75**_**amplitude**_ **(μV)**	2.5	28	10	5.9	41
**pVEP 15’ P100-N135**_**amplitude**_ **(μV)**	1.6	25	11	6.1	41
**pVEP 60’ P100**_**latency**_ **(ms)**	99	134	112	7.3	41
**pVEP 60’ P100-N75**_**amplitude**_ **(μV)**	1.2	24	7.8	4.2	41
**pVEP 60’ P100-N135**_**amplitude**_ **(μV)**	3.7	32	12	5.7	41
**mERG**_**avg**_ **N1**_**latency**_ **(ms)**	14	19	16	1.2	41
**mERG**_**avg**_ **P1**_**latency**_ **(ms)**	32	53	37	2.9	41
**mERG**_**avg**_ **N1 –P1**_**amplitude**_ **(μV)**	0.3	1.3	0.7	0.2	41

mERG_avg_ = averaged multifocal electroretinogram, N1 = first trough, N75 = trough at 75 ms, N135 = trough at 135 ms, pVEP = pattern visual evoked potential, P1 = first peak, P100 = peak at 100 ms.

**Table 3:** Psychophysical scores on elementary visual function tests and UFOV test of subjects included in the analyses.

	min	max	mean	SD	n
**VA**_**far**_ **best eye (logMAR)**	-0.3	0.0	-0.1	0.1	41
**VA**_**far**_ **worst eye (logMAR)**	-0.3	0.2	0.0	0.1	41
**VA**_**near**_ **best eye (logMAR)**	-0.3	0.1	-0.1	0.1	36
**VA**_**near**_ **worst eye (logMAR)**	-0.2	0.2	0.0	0.1	36
**Crowding intensity (logMAR)**	-0.2	0.3	-0.02	0.1	41
**Contrast sensitivity (Weber contrast)**	2	12	5	2	32
**UFOV1 score (ms)**	13	17	14	0.6	41
**UFOV2 score (ms)**	13	237	30	43	41
**UFOV3 score (ms)**	14	263	88	56	41

n = number of participants, SD = standard deviation, UFOV = useful field of view test, VA = visual acuity

### Relation between elementary visual functions and age

**Fig 2:**
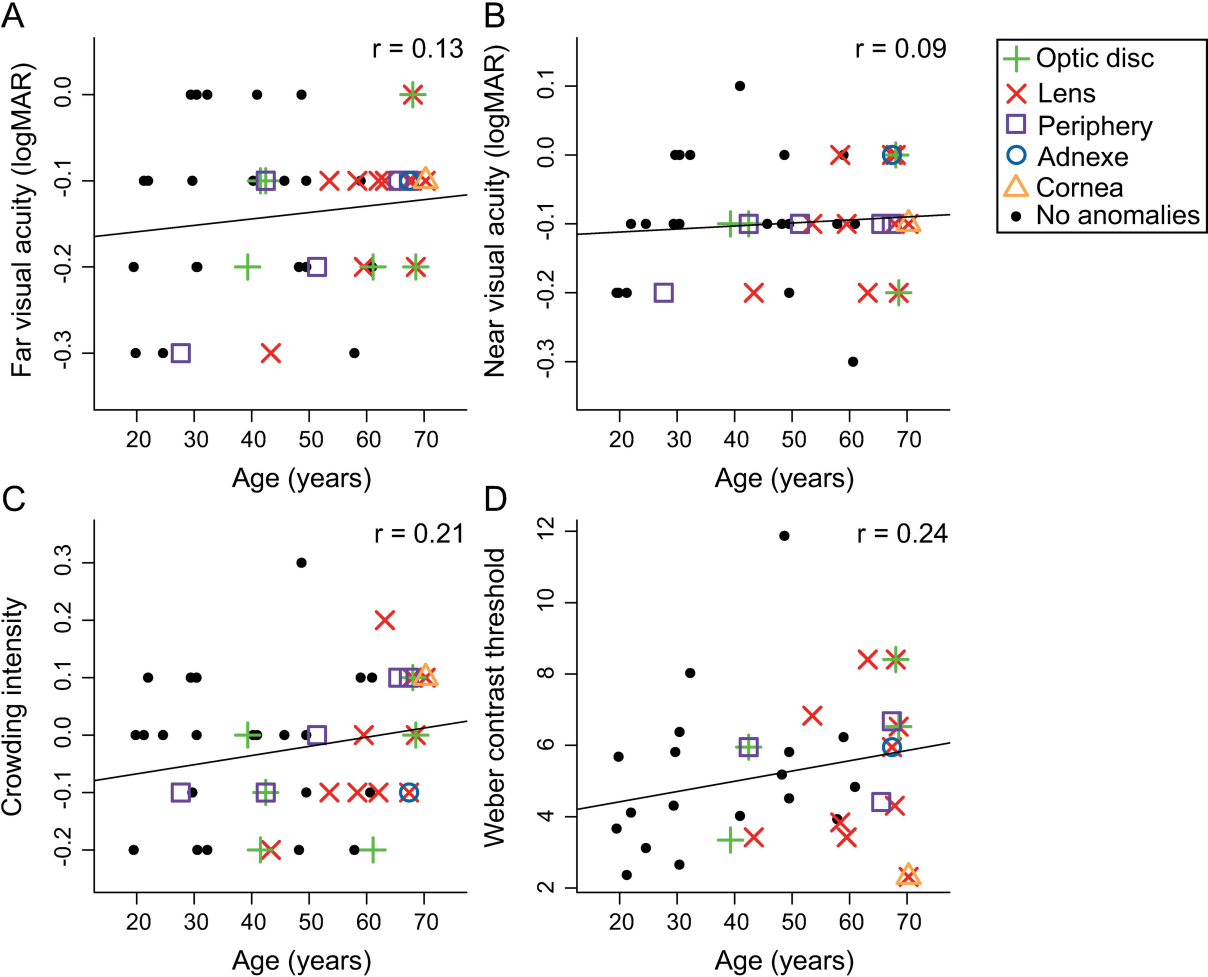
Scatterplots of elementary visual function test scores as a function of age. (A) Far and (B) near visual acuity measured with ETDRS charts at 4 m and 40 cm respectively. (C) Crowding intensity measured with LEA charts at 40 cm. (D) Weber contrast threshold measured with a custom psychophysical task where subjects indicate which of two figures (car or truck similar to UFOV stimuli) is currently presented. Black dots represent participants without any ophthalmic anomalies. Participants with subclinical ophthalmic findings are represented by colored symbols classified by the anatomical structures that were mildly affected. The lines represent the correlation between age and each tested function, none of them were statistically significant.

Fig 2 shows the scatterplots of elementary visual function test scores as a function of age. The different symbols reflect non-pathological anomalies found in each participant’s eyes, classified according to anatomical structures. As Fig 2 shows, subclinical anomalies were more frequently detected in older participants. Nonetheless, we only found small, non-significant correlations between age and the elementary visual function test scores (p-values uncorrected for multiple testing were all larger than 0.19). Thus, despite the non-pathological ophthalmic anomalies in our sample even amongst the older participants, visual acuity, contrast sensitivity and crowding intensity are not significantly related to age.

### Relation between UFOV scores and age

**Fig 3:**
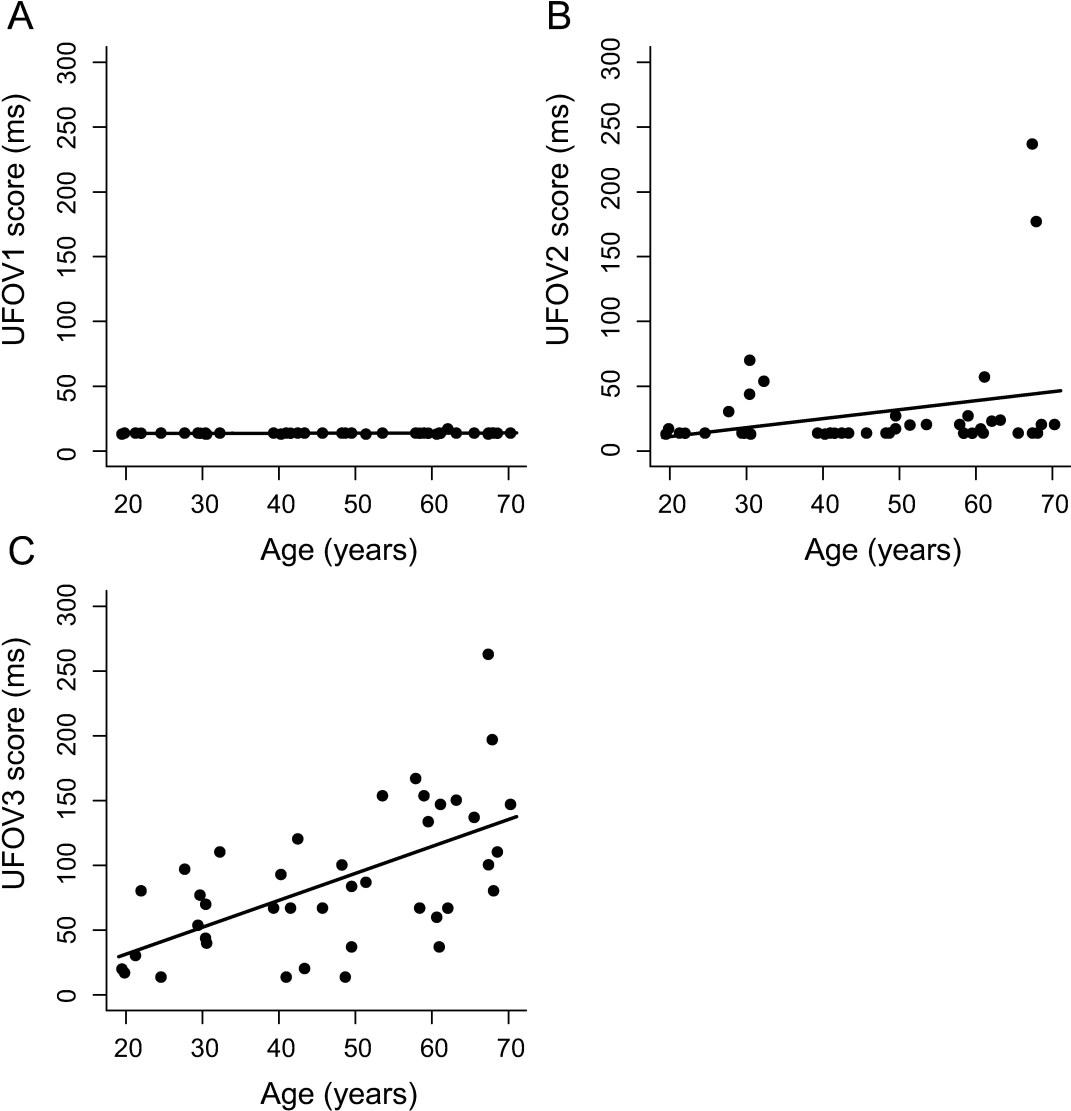
Scatterplots of UFOV scores as a function of age. Presentation time required to respond 75% correct on the (A) first subtest, where subjects indicate which of two figures was presented, (B) second subtest, where subjects indicate which of two figures was presented in the center and where another stimulus was shown in the periphery and (C) third subtest, where the task is similar as for subtest two, but the stimuli are surrounded by distractors, of the commercially available UFOV test. Lines represent the linear predictive effect of age on UFOV subtest scores (*UFOV* ∼ *β*_0_ + *β*_1_ * *age_c_*). UFOV = Useful Field of View.

UFOV1 and UFOV2 scores were in general very low, i.e., performance was good, and showed very little variability (Fig 3). 100% of UFOV1 scores and 85% of UFOV2 scores were lower than 32 ms. UFOV3 scores did not show such a floor effect. We investigated whether UFOV subtest scores were linearly (*UFOV* ∼ *β*_0_ + *β*_1_ * *age_c_*) or quadratically ($UFOV∼\beta_{0}+\beta_{1}*{age}_{c}+\beta_{2a}*{age}_{c}^{2}$) related to age centered on the mean (age_c_) with a series of regression analyses (Fig 3 and Table 4). We did not find a significant effect of age_c_ or age_c_^2^ on UFOV1 or UFOV2 scores. We did find a significant effect of age_c_ on UFOV3 scores; *F*(1, 39) = 21.91, *p*_*FDR*_ < 0.01, R^2^ = 0.36. The bootstrapped regression analysis showed a 95% confidence interval of 0.41–0.74 for standardized β_age_. Adding age_c_^2^ to the model did not improve the fit significantly (*F*_*change*_ (1,38) = 1.17, *p*_*FDR*_ = 0.60, *ΔR*^*2*^ = 0.02). Results of the regression analyses of the total (i.e., summed) UFOV scores are reported in the supplemental material (S2 Fig & S1 Table).

**Table 4:** Results of regression analyses and comparisons of the univariate regression analyses with the multiple regression analyses for each UFOV subtest.

	Model	Fit statistics	p-value	R^2^ or *Δ*R^2^	β’_age_	β’_pred_
**UFOV1**	Linear	F(1, 39) = 1.34	0.25	0.03	0.18	
	Quadratic	F_change_ (1,38) < 0.001	0.98	*Δ*R^2^ < 0.001	0.18	-0.005
	VA_far_ best eye	F_change_ (1,38) = 0.08	0.77	*Δ*R^2^ = 0.002	0.18	0.05
	VA_near_ best eye	F_change_ (1,38) = 0.88	0.36	*Δ*R^2^ = 0.02	0.17	0.15
	Crowding intensity	F_change_ (1,38) = 0.08	0.77	*Δ*R^2^ = 0.002	0.19	-0.05
	Contrast sensitivity	F_change_ (1,38) = 0.20	0.66	*Δ*R^2^ = 0.005	0.20	-0.07
**UFOV2**	Linear	F(1, 39) = 2.77	0.10	0.07	0.26	
	Quadratic	F_change_ (1,38) = 2.45	0.13	*Δ*R^2^ = 0.06	0.32	0.25
	VA_far_ best eye	F_change_ (1,38) = 0.38	0.54	*Δ*R^2^ = 0.009	0.24	0.1
	VA_near_ best eye	F_change_ (1,38) <0.001	0.98	*Δ*R^2^ < 0.001	0.26	-0.003
	Crowding intensity	F_change_ (1,38) = 0.72	0.40	*Δ*R^2^ = 0.02	0.23	0.13
	Contrast sensitivity	F_change_ (1,38) = 0.28	0.60	*Δ*R^2^ = 0.007	0.24	0.08
**UFOV3**	Linear	F(1, 39) = 21.91	< 0.001	0.36	0.60	
	Quadratic	F_change_ (1,38) = 1.17	0.29	*Δ*R^2^ = 0.019	0.64	0.14
	VA_far_ best eye	F_change_ (1,38) = 0.01	0.92	*Δ*R^2^ < 0.001	0.60	0.01
	VA_near_ best eye	F_change_ (1,38) = 0.06	0.80	*Δ*R^2^ = 0.001	0.60	-0.03
	Crowding intensity	F_change_ (1,38) = 0.09	0.74	*Δ*R^2^ = 0.002	0.61	-0.04
	Contrast sensitivity	F_change_ (1,38) = 0.082	0.78	*Δ*R^2^ = 0.001	0.59	0.04

The model column indicates which model’s results are described. For the linear model, which only contains age_c_ as a predictor, the results of the model fit itself are given whereas for every other model, the results of the comparison are reported together with the standardized effect sizes for both age_c_ and the additional quadratic or elementary visual function variable. P-values are not corrected for multiple comparisons. β’_age_ = standardized effect size of age_c_, β’_pred_ = standardized effect sizes of the additional predictor, i.e. age_c_^2^ or an elementary visual function variable, R^2^ = explained variance, *Δ*R^2^ = difference explained variance between model with and without additional quadratic or elementary visual function variable, VA_far_ = far visual acuity, VA_near_ = near visual acuity.

### Relation between UFOV scores and elementary visual functions

**Fig 4:**
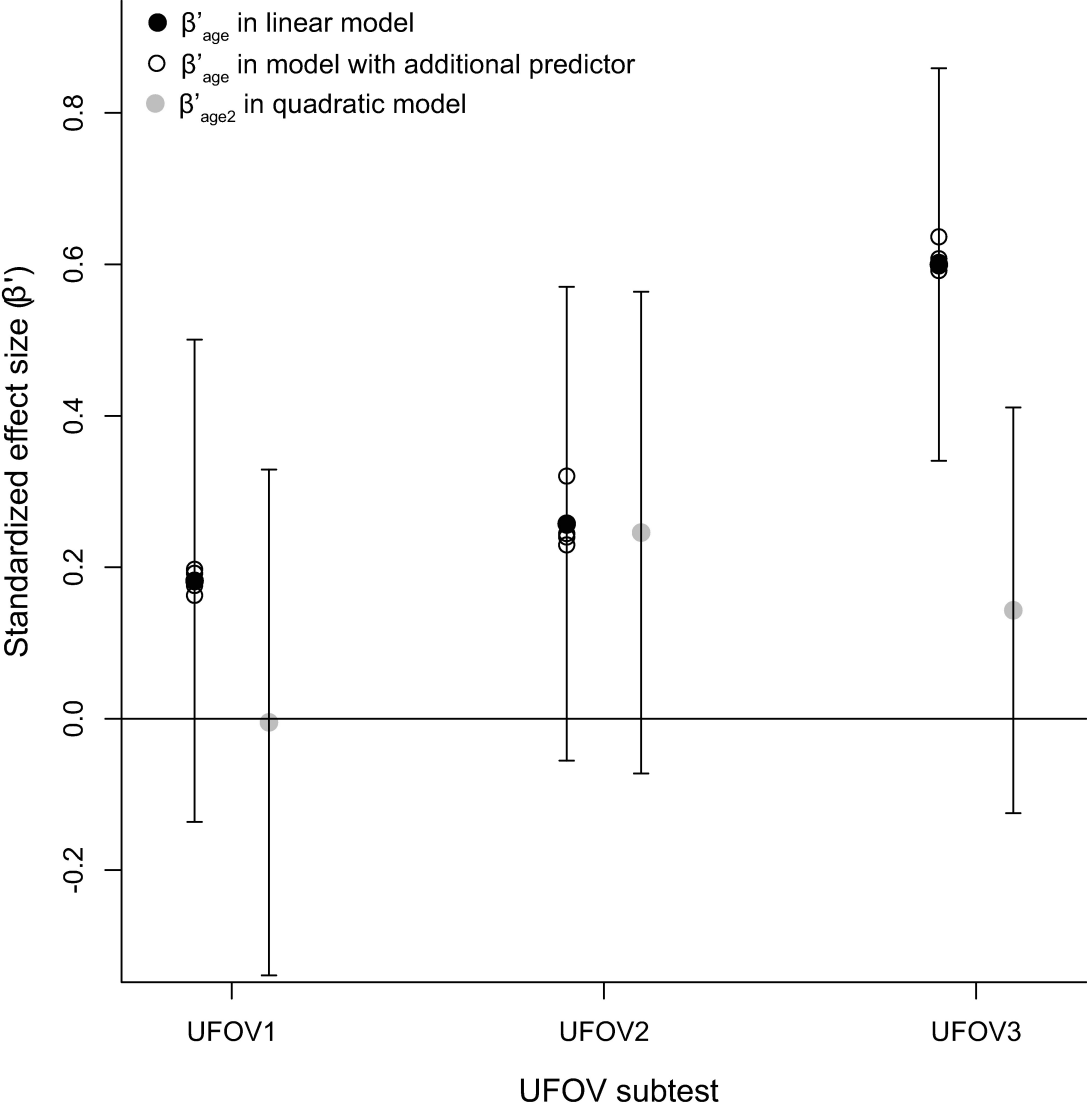
Estimated standardized effect sizes (β’) of age_c_ and age_c_^2^. The black and grey filled circles and their 95% confidence intervals represent the standardized estimates of the effects of age_c_ and age_c_^2^ in the linear and quadratic regression models, respectively. The open circles represent the standardized estimates of age_c_ in the quadratic model and in models with additional elementary visual function predictors, i.e., near and far visual acuity measured with ETDRS charts measured at 4 m and 40 cm respectively, crowding intensity measured with LEA charts at 40 cm and contrast sensitivity represented by the Weber contrast threshold measured with a custom psychophysical task where subjects indicate which of the two figures (car or truck similar to UFOV stimuli) is currently presented. β’ = estimated standardized effect size, UFOV = Useful Field of View.

To investigate whether the elementary visual functions, i.e., near and far visual acuity, crowding intensity and contrast sensitivity, would explain the same variance in UFOV scores as age, we repeated the regression analyses of UFOV scores as a linear function of age and added the elementary visual function variables as individual covariates (*UFOV* ∼ *β*_0_ + *β*_1_ * *age_c_* + *β*_2*b*_ * *var_elementary–visual–function_*). None of the elementary visual function variables improved the regression model significantly (Table 4). In addition, the effects of age remained almost the same (Fig 4).

## Discussion

We investigated age-effects on UFOV performance throughout adulthood in a group of healthy, normally sighted subjects aged 19.5 to 70.3 years. All subjects underwent thorough ophthalmological screening to exclude any previously unknown visual disorders. We also examined non-pathological changes in elementary visual functions, i.e., near and far visual acuity, contrast sensitivity and crowding intensity, and whether these could explain age-related changes in UFOV performance.

UFOV1 and UFOV2 performance did not change throughout the adult population. This is possibly due to the floor effect we observed in the scores. That is, most participants reached the best possible score on these two tasks which caused the variation to be very small. UFOV3 performance declined linearly throughout adulthood. The same was true for the sum of UFOV scores (see S2 Fig and S1 Table), but due to the good UFOV1 and UFOV2 performance independent of the subjects’ ages, this result was mainly driven by the UFOV3 results. Although we identified some mild, non-pathological anomalies in our subjects’ eyes, we found no relationship between age and elementary visual functions or between elementary visual functions and UFOV performance. In addition, they did not explain the age-related decline we found in UFOV3 performance.

It is somewhat surprising that we did not find many of the previously demonstrated associations. We only found linear relationships between age and UFOV3 scores, but not between age and elementary visual functions, between age and UFOV2 or UFOV1, or between elementary visual functions and UFOV scores. Due to the limited sample size, our power may have been too low to detect such relationships. Power analyses show that the power to detect correlations between age and UFOV subtests was 0.22, 0.81 and 0.89 for UFOV1, UFOV2 and UFOV3, respectively (S2 Table). This suggests that for UFOV1 we may indeed have lacked power to detect a correlation. Previously reported correlations between UFOV2 and age, however, should have been large enough to detect in this study.

The fact that we did not find some of the previously reported correlations could also be related to the high performance our subjects showed on most tasks. For example, UFOV scores are about 2–4 times lower than the normative values of subjects aged 65 years and older provided by Edwards et al. [7]. In addition, visual acuity of our subjects, including those older than 65 years, was at most 0.0 and 0.1 logMAR for far and near vision, respectively, which is better than many other studies that investigated UFOV performance. The high visual acuities can possibly be attributed to the strict inclusion criteria and thorough ophthalmic screening that we applied. Previous studies have relied on self-report or a lower cut-off visual acuity score or did screen their participants but included patients nonetheless. Other studies that applied stricter exclusion criteria and performed a thorough ophthalmic screening similar to ours, showed that the visual acuity of subjects without visual pathology is indeed usually better than 0.0 logMAR even in older subjects up to 75 years [22,23]. In fact, the size of 0.0 logMAR or 1 arcminute as a norm was once chosen based on its physical size, to be replicated across clinics and research labs [44,45]. Snellen explicitly stated that this norm does not represent the mean or maximal visual acuity ([45] p. 6) of a population and that letters of this size are “clearly distinguished by a normal eye” ([46] p. 2). Thus, we do believe our sample is a good representation of healthy aging adults albeit somewhat younger than other studies on UFOV performance. Importantly, this suggests that the current normative UFOV values may be too high to be sensitive to small deviations as a result of higher-order visual or cognitive functioning in adults up to 70 years with normal elementary visual functions. However, this should be verified in a larger sample.

### Decline UFOV3 performance

As opposed to UFOV1 and UFOV2, we did find an age-related decline in UFOV3 performance which was of similar magnitude to that found in other studies [7,10,15,47–49]. Although the task instructions for UFOV2 and UFOV3 are essentially the same, the presence of distractors changes the nature of the task considerably. Although both tasks require the subject to indicate the location of the peripheral stimulus, due to its high contrast and isolated presentation in UFOV2, the stimulus clearly pops out. In UFOV3 on the other hand, the presence of distractors that are approximately the same size and contrast, prevents a pop-out effect and requires the target stimulus to be recognized. It may therefore be influenced by both peripheral acuity and crowding. The fact that none of our elementary visual functions, including visual acuity and crowding intensity could statistically explain the age-related decline in UFOV3 performance may be because we measured them centrally. Both visual acuity and crowding are worse when stimuli are presented peripherally instead of centrally [50]. Whether or not these effects increase with age is unclear since contradicting results have been reported [28,51]. In addition to visual functions, the age-related decline of UFOV3 performance could also be related to higher cognitive processes. Previous research has shown many correlations between UFOV3 performance and other cognitive tasks including general cognition, executive functioning and change detection (e.g., [10], for a summary see [11]). Although we excluded participants who reported the presence of a neurological disorder, we did not screen the general cognitive abilities of the subjects explicitly. Since cognitive decline commonly occurs with aging, our sample may have included subjects who were unaware of the presence of a cognitive disorder with a gradual onset. However, none of the included subjects showed obvious signs of cognitive decline during their visit, or reported them during an interview that covered medical history, participation in society, and family history.

### Elementary visual functions

We measured elementary visual functions that were previously related to UFOV performance, i.e., near and far visual acuity, crowding intensity and contrast sensitivity. We found that these functions remain quite good at older ages if visual pathologies are absent. As mentioned above, we found maximal visual acuities of 0.0 and 0.1 logMAR for far and near distances respectively. Unlike previous studies, we found no relation between visual acuity and age [22,23,25,52]. Although acuities were high on both tests, variances do not seem to be restricted, as opposed to the UFOV1 and UFOV2 scores. Our sample size may have been too small to detect a correlation due to a lack of power. However, Elliott et al. reported a relationship between far visual acuity and age with an r^2^ of 0.34 [22]. Such an effect size should be large enough to detect in our sample, as our power was 0.99 (S2 Table) Gittings and Fozard reported declines in near visual acuity [25]. Although their participants also underwent a physical exam, the authors noted that a focused ophthalmological examination might have revealed more visual pathologies. Thus, perhaps a correlation between age and visual acuity exists also in the absence of pathologies, but its effect size may be significantly smaller than those reported in the past. In addition, we found that most participants have a Weber contrast threshold lower than 8.5. Again, we found no age-related effect on contrast sensitivity. Although age-related declines in contrast sensitivity have been reported in the past, this is mainly the case for spatial frequencies of at least 2 cycles/degree [27]. In our custom-made contrast task, we used the same stimuli as the UFOV task to make the two more comparable. These stimuli consisted mainly of spatial frequencies below 1.0 cycles/degree. The effect of age may therefore have been reduced. Last, we measured crowding intensity, i.e., the difference in near binocular vision measured with a crowded chart and an uncrowded chart. We found that this measure varied around zero with one or two lines difference in a visually healthy sample. We found no significant relationship with age. Although previous studies have also included subjects of various ages, they were interested in the detrimental effect of presbyopia and did not correct for this condition [53]. Thus, to our knowledge, we are the first to investigate foveal crowding throughout adulthood in a group without visual pathology and optimally corrected for test distance.

### Conclusion

Our results show that visual functioning remains high as long as there is no obvious ocular pathology. This suggests that underperformance on the UFOV tests might be an early warning sign for the presence of ocular pathology. Subjects may be unaware of their pathologies and self-report is therefore insufficient. Instead, subjects should receive a thorough ophthalmological examination to confirm the absence of ocular pathologies. If ocular diseases are indeed absent, visual functioning may stay relatively stable with age or the decline may be smaller than reported in the past. Performance on the third UFOV subtest does decrease with age, even in the absence of obvious pathology. This decrease is unrelated to near or far visual acuity, foveal crowding or contrast sensitivity.

## Supporting information

S1 Fig Frequency power spectra of stimuli used in the contrast task and a Landolt C optotype. Top row shows the stimuli used in the contrast sensitivity task and a Landolt C with an opening size equal to the midline of the car window for comparison. The bottom row shows the power in the stimuli and optotype for spatial frequencies between 0 and 30 cycles per degree. Most power for the car and truck lies in frequencies below 1.0 cycles/degree.
(TIF)

S2 Fig Relationship between total UFOV scores and age. A) Scatterplot of total UFOV scores as a function of age. The total UFOV score constitutes the sum of the presentation times required to respond 75% correct on the three UFOV subtests. Total UFOV scores were 41–513 ms, with a mean of 131 ± 89 ms. The line represents the linear predictive effect of age on UFOV subtest scores (*UFOV* ∼ *β*_0_ + *β*_1_ * *age_c_*). We found a significant effect of age_c_ on total UFOV scores (*F* (1,39) = 12.93, *p*_*FDR*_ < 0.01, *R*^*2*^ = 0.23). B) Estimated standardized effect size (β’) of age_c_ and age_c_^2^. The black and grey filled circles and their 95% confidence intervals represent the standardized estimates of the effects of age_c_ and age_c_^2^ in the linear and quadratic regression models, respectively. The open circles represent the standardized estimates of age_c_ in the quadratic model and in models with additional elementary visual function predictors, i.e., near and far visual acuity measured with ETDRS charts measured at 4 m and 40 cm respectively, crowding intensity measured with LEA charts at 40 cm and contrast sensitivity represented by the Weber contrast threshold measured with a custom psychophysical task where subjects indicate which of two figures (car or truck similar to UFOV stimuli) is currently presented. β’ = estimated standardized effect size, UFOV = Useful Field of View.
(TIF)

S3 Fig Forest plots of Pearsons’ correlation coefficients between UFOV (summed) scores and age. Correlations are shown as points with their 95% confidence intervals and categorized to UFOV subtests. Values are listed under *r[ci]*. We used a random effects analysis to estimate the true underlying effect size and its 95% confidence interval which is depicted here as the polygon using R version 3.1.2 [38] with the ‘metafor’ package [54](for more details, see [11]). [55].
(TIF)

S1 Table Results of regression analyses of the total UFOV scores. Results of regression analyses and comparisons of the univariate regression analyses with the multiple regression analyses for the total, i.e., summed UFOV scores. The model column indicates which model’s results are described. For the linear model, which only contains age_c_ as a predictor, the results of the model fit itself are given whereas for every other model, the results of the comparison are reported together with the standardized effect sizes for both age_c_ and the additional quadratic or elementary visual function variable. P-values are not corrected for multiple comparisons.
(DOCX)

S2 Table Results of power analyses. Results of power analyses of previously reported relationships between UFOV scores and age and between far visual acuity and age. We estimated correlations between the (summed) UFOV scores and age with a random effects meta-analysis of Pearson’s correlation coefficients reported in articles included in [11], using the ‘metafor’ package version 1.9–8 [54] in R (S3 Fig). The results of a regression analysis of the relationship between age and far visual acuity are reported in [22], below we show the square root of the reported r^2^. We calculated the power with the ‘pwr’ package version 1.2–1 [56] using a sample size of 41 and significance level of 0.05 (two-tailed).
(DOCX)
