# Supplementary figures and images for: Useful field of view test performance throughout adulthood in subjects without ocular disorders
Source: PLoS One. 2018 May 1;13(5):e0196534. doi: 10.1371/journal.pone.0196534 (PMC5929545; doi:10.1371/journal.pone.0196534)

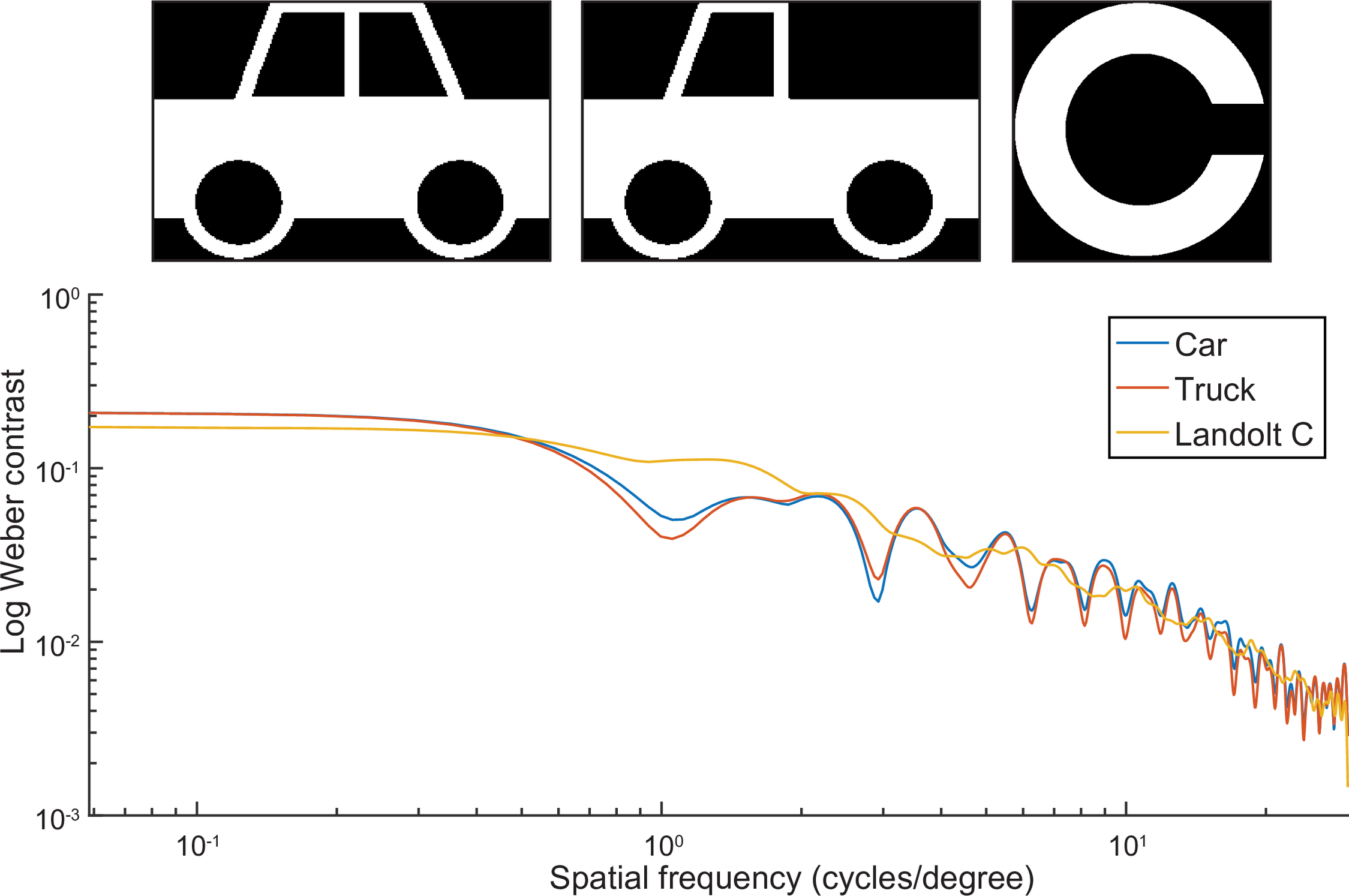

Supplement: S1 Fig — Top row shows the stimuli used in the contrast sensitivity task and a Landolt C with an opening size equal to the midline of the car window for comparison. The bottom row shows the power in the stimuli and optotype for spatial frequencies between 0 and 30 cycles per degree. Most power for the car and truck lies in frequencies below 1.0 cycles/degree. (TIF) [file pone.0196534.s001.tif]

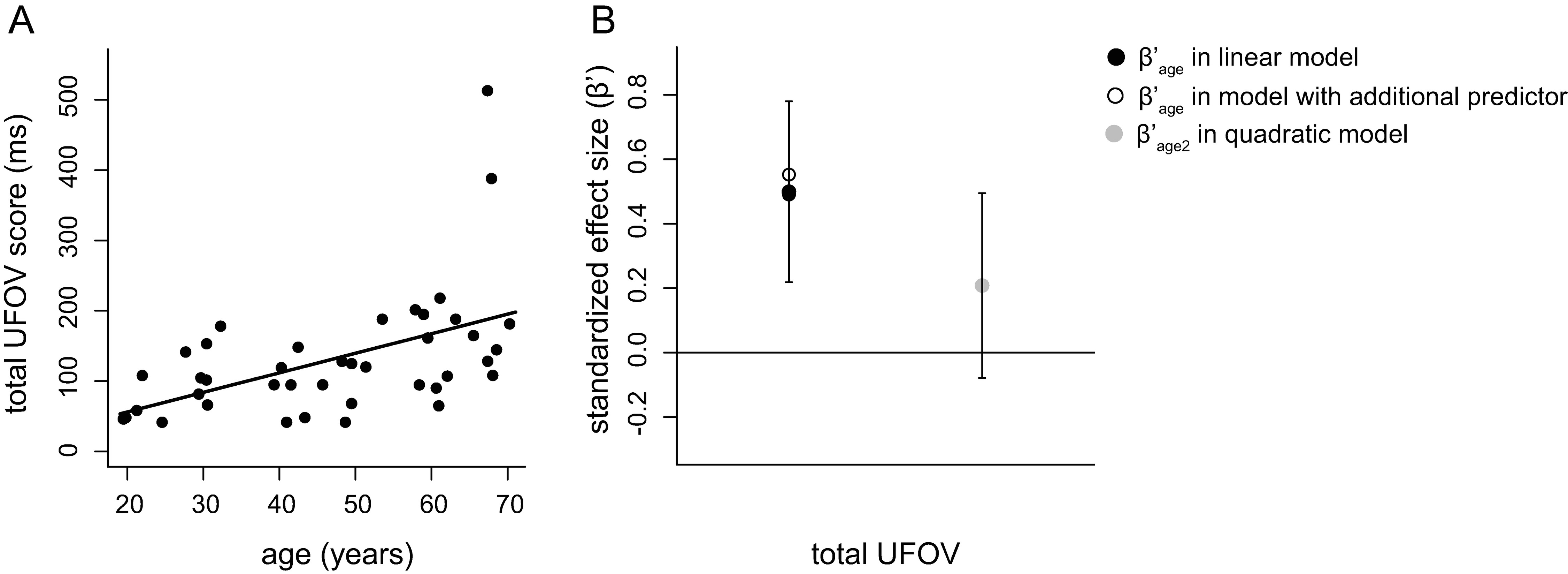

Supplement: S2 Fig — A) Scatterplot of total UFOV scores as a function of age. The total UFOV score constitutes the sum of the presentation times required to respond 75% correct on the three UFOV subtests. Total UFOV scores were 41–513 ms, with a mean of 131 ± 89 ms. The line represents the linear predictive effect of age on UFOV subtest scores (UFOV ∼ β0 + β1 * agec). We found a significant effect of agec on total UFOV scores (F (1,39) = 12.93, pFDR < 0.01, R2 = 0.23). B) Estimated standardized effect size (β’) of agec and agec2. The black and grey filled circles and their 95% confidence intervals represent the standardized estimates of the effects of agec and agec2 in the linear and quadratic regression models, respectively. The open circles represent the standardized estimates of agec in the quadratic model and in models with additional elementary visual function predictors, i.e., near and far visual acuity measured with ETDRS charts measured at 4 m and 40 cm respectively, crowding intensity measured with LEA charts at 40 cm and contrast sensitivity represented by the Weber contrast threshold measured with a custom psychophysical task where subjects indicate which of two figures (car or truck similar to UFOV stimuli) is currently presented. β’ = estimated standardized effect size, UFOV = Useful Field of View. (TIF) [file pone.0196534.s002.tif]

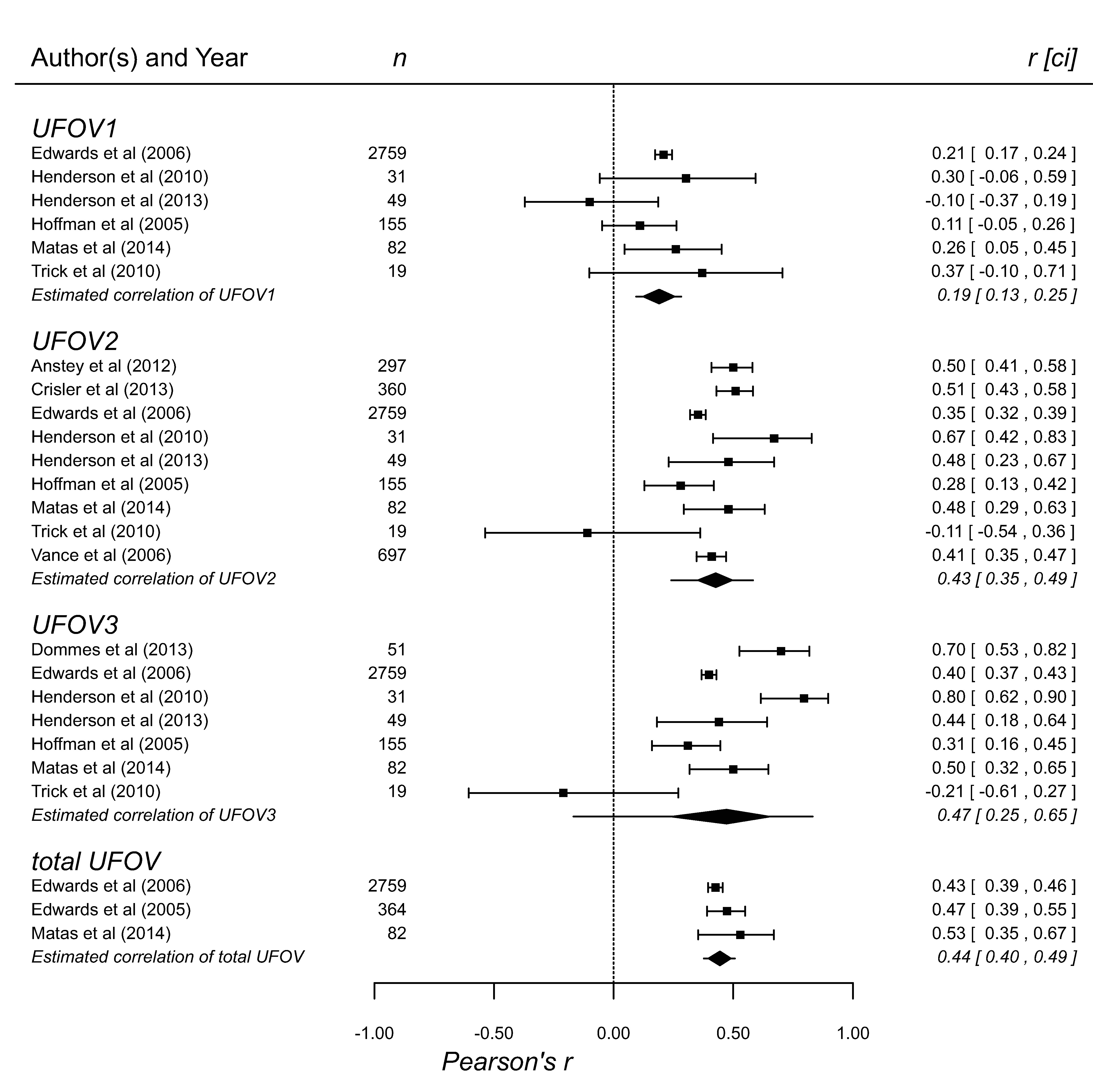

Supplement: S3 Fig — Correlations are shown as points with their 95% confidence intervals and categorized to UFOV subtests. Values are listed under r[ci]. We used a random effects analysis to estimate the true underlying effect size and its 95% confidence interval which is depicted here as the polygon using R version 3.1.2 [38] with the ‘metafor’ package [54](for more details, see [11]). [55]. (TIF) [file pone.0196534.s003.tif]
