# Supplementary material for: Useful field of view test performance throughout adulthood in subjects without ocular disorders
Source: PLoS One. 2018 May 1;13(5):e0196534. doi: 10.1371/journal.pone.0196534 (PMC5929545; doi:10.1371/journal.pone.0196534)
Supplement: S1 Table — Results of regression analyses and comparisons of the univariate regression analyses with the multiple regression analyses for the total, i.e., summed UFOV scores. The model column indicates which model’s results are described. For the linear model, which only contains agec as a predictor, the results of the model fit itself are given whereas for every other model, the results of the comparison are reported together with the standardized effect sizes for both agec and the additional quadratic or elementary visual function variable. P-values are not corrected for multiple comparisons. (DOCX) [file pone.0196534.s004.docx]

| **Model** | **Fit statistics** | **p-value** | **R^2^ or *Δ*R^2^** | **β’_age_** | **β’_pred_** |
| --- | --- | --- | --- | --- | --- |
| Linear | F(1, 39) = 12.93 | <0.001 | 0.25 | 0.50 |  |
| Quadratic | F_change_ (1,38) = 2.16 | 0.15 | *Δ*R^2^ = 0.04 | 0.55 | 0.21 |
| VA_far_ best eye | F_change_ (1,38) = 0.15 | 0.70 | *Δ*R^2^ = 0.003 | 0.49 | 0.06 |
| VA_near_ best eye | F_change_ (1,38) = 0.02 | 0.88 | *Δ*R^2^ < 0.001 | 0.50 | -0.02 |
| Crowding intensity | F_change_ (1,38) = 0.08 | 0.78 | *Δ*R^2^ = 0.002 | 0.49 | 0.04 |
| Contrast sensitivity | F_change_ (1,38) = 0.2 | 0.66 | *Δ*R^2^ = 0.004 | 0.49 | 0.06 |

β’_age_ = standardized effect size of age_c_, β’_pred_ = standardized effect sizes of the additional predictor, i.e. age_c_^2^ or an elementary visual function variable, R^2^ = explained variance, *Δ*R^2^ = difference explained variance between model with and without additional quadratic or elementary visual function variable, VA_far_ = far visual acuity, VA_near_ = near visual acuity.
