# Supplementary material for: Useful field of view test performance throughout adulthood in subjects without ocular disorders
Source: PLoS One. 2018 May 1;13(5):e0196534. doi: 10.1371/journal.pone.0196534 (PMC5929545; doi:10.1371/journal.pone.0196534)
Supplement: S2 Table — Results of power analyses of previously reported relationships between UFOV scores and age and between far visual acuity and age. We estimated correlations between the (summed) UFOV scores and age with a random effects meta-analysis of Pearson’s correlation coefficients reported in articles included in [11], using the ‘metafor’ package version 1.9–8 [54] in R (S3 Fig). The results of a regression analysis of the relationship between age and far visual acuity are reported in [22], below we show the square root of the reported r2. We calculated the power with the ‘pwr’ package version 1.2–1 [56] using a sample size of 41 and significance level of 0.05 (two-tailed). (DOCX) [file pone.0196534.s005.docx]

| Test | Effect size (*r*) | Power |
| --- | --- | --- |
| UFOV1 | 0.19 | 0.22 |
| UFOV2 | 0.43 | 0.81 |
| UFOV3 | 0.47 | 0.89 |
| Total UFOV | 0.44 | 0.85 |
| VA_far_ | 0.58 | 0.99 |

UFOV = Useful Field of View, VA_far_ = far visual acuity
